# The role of shared decision-making in improving adherence to pharmacological treatments in patients with schizophrenia: a clinical review

**DOI:** 10.1186/s12991-020-00293-4

**Published:** 2020-08-05

**Authors:** Andrea Fiorillo, Stefano Barlati, Antonello Bellomo, Giulio Corrivetti, Giuseppe Nicolò, Gaia Sampogna, Valentina Stanga, Franco Veltro, Giuseppe Maina, Antonio Vita

**Affiliations:** 1 Department of Psychiatry, University of Campania “L. Vanvitelli, Largo Madonna delle Grazie, Naples, Italy; 2 Department of Mental Health and Addiction Services, ASST Spedali Civili, Brescia, Italy; 3 Department of Clinical and Experimental Sciences, University of Brescia, Brescia, Italy; 4 Department of Clinical and Experimental Medicine, Psychiatric Unit, University of Foggia, Foggia, Italy; 5 Department of Mental Health, Salerno, Italy; 6 Department of Mental Health Colleferro, ASL Roma G, Tivoli, Italy; 7 Mental Health Department of Campobasso, Campobasso, Italy; 8 Department of Neuroscience, University of Torino, Turin, Italy

**Keywords:** Shared decision-making, Recovery, Empowerment, Adherence, Schizophrenia, Long-acting injectable antipsychotics

## Abstract

Shared decision-making (SDM) is a process in which the doctor provides clear and complete medical information to patients about their treatment, and patients provide information on his/her preferences. Patients and clinicians bring different, but equally important, knowledge to the decision-making process. Through the adoption of SDM, it should be possible to overcome the barriers that hinder the acceptance of long-acting injectable antipsychotics (LAIs) by patients, and often also by psychiatrists. The present paper is a critical appraisal of recent literature on the impact of SDM in improving adherence to pharmacological treatments and in implementing the use of LAIs in the treatment of patients with schizophrenia. SDM is recognized as a promising strategy to improve collaboration between clinicians and patients in achieving recovery. When considering drug treatments, clinicians must evaluate the patient’s preferences, expectations and concerns towards the development of a personalized treatment strategy. Moreover, an active involvement in the decision process could reduce the patient’s perception of being coerced into the use of LAIs. Involving patients in the choice of therapy is not sufficient to increase pharmacological adherence if, at the same time, there is no constant work of comparison and communication with the reference psychiatric team. SDM can be particularly effective for LAI prescription, since patient can have prejudices and unjustified fears related to the LAI formulation, which the doctor must resolve.

## Background

In accordance with its most common acceptation, clinical decision-making has been traditionally practiced as a one-way evidence-based process on behalf of the clinicians alone: a “contextual, continuous, and evolving process where data are gathered, interpreted, and evaluated by the clinician in order to select an evidence-based choice of action” [﻿^1^﻿]. In recent years, the process has been gradually remodeled to become a more patient-inclusive approach letting the voice of those directly affected by the decisions to be heard [﻿2﻿]. In such spirit, the clinician empowers the patient to take part on his/her own treatment strategy by providing the patient clear and exhaustive medical information, while listening to the patient’s preferences and priorities and facilitating the patient’s evaluations towards a balanced reasoned decision. It is a negotiation between the clinician and patient taking place for achieving a shared decision [3].

In the general medical setting, the type of decision-making is influenced by the balance achieved in the clinician–patients relationship and positions itself along a continuum, ranging from the paternalistic (clinician-led or passive style), through shared decision-making (SDM), up to the patient-led active style (also known as informed style) [4]. The unfolding and outcomes of such process depend on the variables informing the process related to: (a) the patients, and their personal attitudes/preferences, cognitive symptoms, levels of self-stigma; (b) the healthcare professionals, in terms of years of professional experience and professional role [5﻿]; (c) contextual and unspecific factors, such as communication skills (verbal and non-verbal behaviors), setting, therapeutic alliance, and others [﻿1﻿, 6].

With specific reference to the SDM style, studies have demonstrated that it has a positive impact on the patient’s levels of satisfaction and adherence to treatments, as well as on his/her quality of life and empowerment [7]. This has been especially highlighted in the case of patients with severe mental disorders who report a greater desire of being involved in clinical decision-making and a need to have a say in the process of care, as compared to individuals receiving assistance for other medical conditions [8–11]. Patients and clinicians bring different—but equally important—knowledge and expertise to the decision process, which need to be integrated [12]. When patients are involved in choices about their own health and care, they ponder options carefully and are most likely to appreciate the value of proposed treatment, to agree to treatment with a favorable attitude. In fact, shared process has proven to increase adherence to the prescribed treatment and improve long-term outcomes. Furthermore, this has also translated in more efficient allocation of healthcare resources [13–15].

These latter aspects related to adherence are especially relevant in the setting of schizophrenia and psychotic disorders, where adherence to pharmacological treatments is frequently far from optimal and represents the main cause of relapse [16–18] and hospitalizations [19, 20]. The advent of new long-acting injectable antipsychotics (LAIs) had appeared to overcome the issue of poor adherence [21–24﻿], but did not solve the widespread lack of adherence, as these drugs still remain largely underutilized. Currently, a number of studies have proven the effectiveness, safety and tolerability of LAIs [25], yet recommendations on their use in the clinical routine care differ from one guideline to another, and their current use is still limited despite their proved efficacy on long-term patient management [26﻿]. In some cases, the use of LAIs is recommended only for patients with frequent relapses and/or poor adherence [27﻿] and for those preferring LAIs over oral therapy [28]. The Canadian Schizophrenia Guidelines suggest that early use of LAIs in the management of schizophrenia should be advocated, without limiting its use to those patients for whom non-adherence is a concern [﻿26﻿]. Moreover, only the French Association for Biological Psychiatry and Neuropsychopharmacology expert consensus guidelines propose LAIs to patients upon their first episode of psychosis and only after an adequate patient-informed consensus [29]. In the United States only 15–28% of patients with schizophrenia receive a LAI [30, 31]. In Europe only 40% of clinicians would use LAIs for treating first-episode psychosis [32], while a large portion of them tend to use LAIs only in the case of patients with long-term disease and poor compliance [﻿33﻿, 34]. Finally, the routine use of LAIs is delayed by other issues such as the patients’ attitude towards the drug (the fear of needles or of side-effects), the perception that LAIs are imposed on them in a punitive and coercive manner, or as result of a previous negative experience with LAIs, or in consideration of the negative perception of LAIs by their family members. Resolving such resistances hence could lead to more patients benefitting from LAIs.

In such scenario, SDM is certainly an interesting approach to achieve greater knowledge of LAIs and acceptance on behalf of patients and families [﻿35﻿, ﻿^36^﻿]. Although SDM has been repeatedly advocated as the preferred style in routine clinical practice, its dissemination in ordinary settings is not satisfying. According to the CEDAR multicenter study, SDM is adopted only when patients present a good level of personal and social functioning and when professionals have a long-term experience in working in the mental health field [﻿5﻿]. It is thus necessary to develop strategies for improving the adoption of SDM in clinical routine care.

The present clinical review aims to provide an update on the available interventions for improving SDM in routine care and to discuss the positive role of SDM style in improving adherence to pharmacological treatments and in the definition of a personalized treatment plan for patients with schizophrenia, particularly in switching pharmacological regimen from oral to LAI formulations.

## Methods

**Fig. 1:**
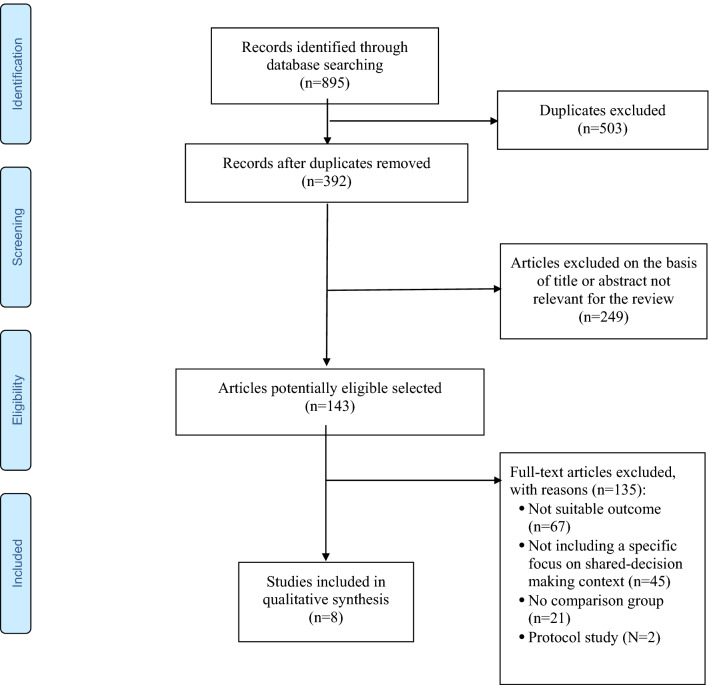
PRISMA flow-chart

The present review was based on search of key words “shared decision making”, “intervention”, “schizophrenia”, “psychosis”, “schizophrenia spectrum disorder” matched with “adherence”, “intervention”, “training”, “long-acting injectable antipsychotic”, “LAI” in the main databases MEDLINE, ISI Web of Knowledge—Web of Science Index, Cochrane Reviews Library and PsychoINFO. The search considered recent papers published between 2009 and 2019, as publications from previous years had already been covered by Duncan et al. [37]. The search was limited to papers in English and published in peer-reviewed journals. The references’ lists of all included papers have been carefully searched in order to identify further papers relevant for the review. In case of discrepancies between the two evaluators in the study selection, these were solved through discussion with a senior expert researcher. Finally, recent international guidelines on the management of patients with schizophrenia were searched as well. Randomized-controlled trials, quasi-experimental studies, and pilot studies were included in the review in order to provide an updated overview on the topic, as extensive as possible. The selection process of the articles included is illustrated in Fig. 1.

## Results

The main features of the included studies [35, 38–44] are summarized in Table 1.

**Table 1:** Main features of the included studies

Author (Country), Year	Study design	Sample size and setting	Diagnosis	Inclusion criteria	Intervention	Outcome
Hamann et al. (Germany), [38]	Pilot study with random allocation	*N* = 61 patients (shared decision making training, *N* = 32; control condition, *N* = 29); acute psychiatric ward	Schizophrenia, schizoaffective disorder	Patients’ age 18–60 years	Shared decision-making training: five 1-h group sessions, including the importance of shared decision-making process, motivational aspects and the use of role-plays Control condition: five-sessions of cognitive training group	Patients receiving the shared decision-making training reported higher participation preferences and increased patients’ desire to have more responsibility in treatment decisions, which continued at 6-month follow-up
An et al. (Korea), [39]	Quasi-experimental, non-equivalent pre/post-test design	*N* = 60 (Experimental group, *N *= 29; Control group, *N* = 31); acute psychiatric ward	Schizophrenia or schizoaffective disorder based on DSM-IV-TR criteria	Patients’ age 19 years old or older	SMD training program: consists of 8-weekly group sessions based on the SDM guidelines. The topics include: general information on the program; education on the significance of SDM; communication; expression of patient’s needs and preferences for treatment; understanding the needs of others; coordinating opinions in decision-making situations; demonstrating the SDM in various scenarios; practicing the SDM in real situation Control group: treatment as usual	Patients receiving the SDM training program report an improvement in the levels of self-esteem, problem-solving ability, and quality of life compared to patients allocated in the control group
McCabe et al. (UK), [40]	Cluster randomised control trial	*N* = 72 patients (Experimental group, *N* = 36; Control group, *N* = 36), *N* = 12 psychiatrists; psychiatric out-patient clinics or community mental health services	Schizophrenia or schizoaffective disorder according to ICD-10 criteria	Patients’ age: 18–65 years	TEMPO training: focused to mental health professionals, including the following topics: understanding the patient with psychotic experiences: reflecting on the patient’s experience and the professional and emotional response to psychotic symptoms; communication techniques for working with positive and negative symptoms; empowerment of the patient; involvement in decision-making about medication Control group: treatment as usual	Patients treated by psychiatrists receiving the TEMPO training reported a more positive therapeutic relationship as did psychiatrists
Ramon et al. (United Kingdom), [41]	Naturalistic study, before and after, uncontrolled design	*N* = 47 service users, *N* = 35 care-coordinators and *N* = 12 psychiatrists; community services for adults with long-term mental health problems	Schizophrenia, bipolar disorder and depression	Patients’ age: 18–65 years; in charge at rehabilitation and recovery services for at least 6 months; taking any psychiatric medication for at least 6 months	Training was delivered to separate groups of service users, psychiatrists and care coordinators. The core content was the same for all groups and focused on the process of SDM. Training sessions were delivered at fortnightly or monthly intervals. Training was offered to all psychiatrists and care coordinators who prescribe, monitor or discuss medication with service users	Patients reported a change in decisional conflict and perceptions of practitioners’ interactional style in promoting SDM at the follow-up. A positive impact was found on service users’ and care coordinators confidence to explore medication experience, and group-based training was valued
Ishii et al. (Japan), [42]	Randomized, parallel-group, two-arm, open-label, single-center study	*N* = 24 patients (shared decision making group, *N* = 11; Usual care group, *N* = 13); acute psychiatric ward	Schizophrenia spectrum disorder according to ICD-10 criteria	Patients’ age 16–65 years; no previous psychiatric admission	Shared Decision Making group (SDM): 15–20-min weekly intervention provided during the in-patient stay, consisting of three elements: evaluation of patient’s perceptions of on-going treatments; sharing patients’ and medical staffs’ perceptions on the treatments; shared definition of care plan Usual care group: usual psychiatric inpatient care, which mainly include pharmacological treatments	Patients in the SDM group reported a higher level of satisfaction towards treatments compared to usual care group, while no differences were found in attitude toward medication, treatment continuation and in the levels of global functioning
Hamann et al. (Germany), [43]	Randomized-controlled trial, multicenter study	*N* = 264 (intervention group, *N* = 142; control group, *N* = 122); acute wards of four participating psychiatric hospitals	Schizophrenia, schizoaffective disorder according to ICD-10 criteria	Patients’ age 18–60 years	Shared Decision Making (SDM): 5-session training (60 min/session) addressing patient competencies for SDM, including sessions on motivational and behavioral aspects (e.g., role plays) and on patient–doctor interaction Control group: 5-session of cognitive training, but with no reference to doctor-patient communication	Patients in the SDM group reported an increase in their levels of participation preferences and their wish to take over more responsibility for medical decision. No differences regarding the treatment adherence were found at 6 and 12 months after discharge
Finnerty et al. (USA), [44]	Multicentre study	*N* = 1416 patients (MyCHOIS–CommonGround, *N* = 472; control condition, *N* = 944); 12 Medicaid outpatient clinics	Anxiety disorder, bipolar/depressive disorder, post-traumatic stress disorder, schizophrenia spectrum disorder, sleep–wake disorder, substance-related or addictive disorder	Adult patients served by MyCHOIS–CommonGround clinics between 2011–2014	MyCHOIS–CommonGround: Web-based shared decision-making application on outpatient mental health treatment engagement and on antipsychotic medication adherence Control group: simple random sample of adult Medicaid receiving a mental health clinic service	At one-year follow-up, patients in the MyCHOIS–CommonGround report higher level of engagement in outpatient mental health services and of adherence to antipsychotic medication compared to the control group
Kane et al. (USA), [35]	Randomized controlled trial	*N* = 255 patients; community “real world” mental health clinics	Schizophrenia diagnosis confirmed by SCID-5	Patients’ age: 18-35 years; less than 5 years of antipsychotic lifetime use	Experimental group: to provide LAI treatment with long-acting aripiprazole monohydrate (Aripiprazole Once Monthly). Clinicians received a training course on the role of non-adherence in relapse and hospitalization, effectiveness of LAI antipsychotic, shared decision-making principles, communication strategies Treatment as usual group: defined as the Clinician’s Choice condition	91% of patients accepted at least one LAI antipsychotic during the first 3 months participation to the trial

Ishii et al. [﻿42﻿] developed a training program tailored to patients with schizophrenia during their stay in an acute psychiatric ward. The intervention consists in evaluating the patients’ attitudes on the treatments received, sharing this information with the other clinicians and then identifying a shared plan. Patients in the SDM group reported a higher level of satisfaction towards treatments compared to the usual care group, while no differences were found in attitude toward medication, treatment continuation and the levels of global functioning.

Finnerty et al. [44] proposed interventions based on the use of smartphone applications or “apps”, implemented the MyCHOIS-CommonGround, a decision-making Web-based tool. The “My Collaborative Health Outcomes Information System” (MyCHOIS) is part of a Web-based platform for supporting shared decision-making and quality improvement, developed by the New York State Office of Mental Health. Within the MyCHOIS system, CommonGround application engages patients to complete a CommonGround SDM report prior to the appointments with their doctors. The report evaluates the patient’s perspective on symptoms, functioning, treatment progress and concerns. During the medical examination, patient and clinician review together the report and work towards developing a shared decision. The Web-based tool has been proven to be effective in increasing the level of engagement with the mental health services and in improving adherence to the prescribed treatments.

In Germany, Hamann et al. [38] tested the efficacy of a new shared decision making intervention developed for patients with psychotic disorders. The experimental intervention is tailored for mental health care staff and patients, with the aim of improving communication skills and patient empowerment. The SDM training yielded higher participation preferences and increased patients’ desire to have more responsibility in treatment decisions, which continued at 6-month follow-up.

Another randomized-controlled study carried out in Germany [43﻿] included a SDM-training program for staff members focused on motivational and behavioral aspects. At the end of the study, only short-term differences were found between patients allocated to the experimental group. In 2017, Ramon et al. tested the efficacy of a training program tailored to patients, psychiatrists and care-coordinators [41]. The main training goal was to improve SDM style by using role-play techniques, web-site materials and group discussion. The training program which was dedicated to both service users and practitioners, confirmed the usefulness of SDM on psychiatric medication. In 2015, a group of Korean researchers evaluated the levels of patients’ self-esteem, problem-solving strategies and quality-of-life following a structured SDM training program (eight-session group program for inpatients), which in fact resulted in improvement [﻿39﻿]. In United Kingdom, McCabe et al. [﻿40﻿] developed the TEMPO manualized intervention, addressed to mental health professionals’ and aiming to increase their understanding of patients with psychotic experiences, improve their communication skills while empowering the patient and promoting SDM. Psychiatrists receiving the intervention reported to have a more satisfying therapeutic relationship with their patients.

In US, Kane et al. promoted the PRELAPSE study, which is a randomized-controlled trial including first episode and first-phase patients with schizophrenia allocated to receiving either LAI or treatment as usual [﻿35﻿]. In the study, clinicians attended a training course on the importance of using LAI medication, the role of shared decision-making and on communication strategies for improving patient adherence to pharmacological treatment. At the end of the preliminary recruitment phase [﻿35﻿], authors found that 91% of patients included would have accepted LAIs in the early stage of disease, if this therapeutic choice had been proposed in a supportive way.

## Discussion

In recent years, much effort has been dedicated to find ways of making SDM more effective. The main differences among these experiences are related to the target group (either patients, clinicians, or both), the type of decision-supporting tool (face-to-face or technologically based engagement), and the duration of the follow-up period.

Regarding the target groups, all experimental interventions specifically addressed to patients [38﻿, ﻿^39^, ﻿﻿^44^, ^45^] resulted in a more active behavior during psychiatric consultations within the hospitalization period. However, no clear effects have been found in terms of adherence rate to pharmacological treatments, evidencing the need for adding decision-support tools and stronger communication skills by mental health professionals in order to achieve this objective.

Interestingly, projects specifically addressed to mental health professionals have shown promising results in improving the quality of therapeutic alliance and patient acceptance of pharmacological treatments. In the case of the TEMPO training [﻿40﻿], long-term effectiveness of the intervention was associated with the inclusion of a dedicated session (4-step approach) on SDM in patients with psychosis aimed at changing the patient decision-making style. In the PRELAPSE trial, good short-term results had been achieved by including communication skills training with emphasis on the role of SDM in routine clinical care. Efficacy of the shared approach was also confirmed by the only experience of SDM training course developed for both patients and psychiatrists and care-coordinators by Ramon et al. [﻿41﻿].

Albeit encouraging, however, none of the approaches above could provide key data regarding the stability and the maintenance of the positive effects over time, due to their short-term follow-up. Another issue is the feasibility of these interventions in the clinical routine care, their cost-effectiveness and their usefulness in managing crisis situations.

As regards the web-based tools, engagement through the MyCHOIS–CommonGround website was associated with a higher level of ongoing engagement in out-patient mental health service compared to that of the control group, although no significant differences were found in the adherence rate with antipsychotic medication. Another noticeable advantage was the cost-effectiveness of the intervention which makes it particularly suitable for attaining long-term outcomes in patients with severe mental disorders [46–50﻿]. More recently, the ongoing “Momentum trial” in Denmark [51] is evaluating the effectiveness of a smartphone application in the outcome of treatment consultations, by engaging people with schizophrenia-spectrum disorders and encouraging patient activation and SDM.

SDM is recognized as a promising strategy for enhancing collaboration between clinicians and patients, given the complementary knowledge and expertise of both parties [52]. Patient recovery can be fostered by adopting a SDM style, enhancing empowerment and self-efficacy of patients [52–55]. In turn, SDM has shown its usefulness in improving treatment adherence [﻿^27^﻿]. Konrad et al. [56] found that during clinical encounters, the most frequent decisions taken by clinicians were related to medications and to the severity of symptoms, while patients were rarely involved in the medication choice or given a choice at all.

It is clear that in many cases patients being prescribed antipsychotics would need to understand the advantages and long-term positive impacts on their functional outcome, especially in the case of LAIs where this should be discussed as early as possible [﻿26﻿]. Many mental health care professionals consider the matter of medication to be too sensitive to be discussed with the patient and approached by SDM, too time-consuming for them and somewhat discouraging for the patient (in terms of adverse effects).

From the patients’ viewpoint, patients admit preferring a more directive/paternalistic practitioner style during crisis, but they report also to feel pressured or being persuaded or coerced into accepting pharmacological treatments like LAIs if they fail to take their oral prescribed medication. Clinical decision-making should change on the basis of contextual variables and the style should be tailored to fit patients’ needs and preferences, according to the stage of the illness [2﻿].

However, the adoption of SDM appears useful in the long-term treatment of patients with schizophrenia where medication non-adherence plays an important role in relapse rates, poor outcome, and high costs [﻿36﻿]. As suggested by NICE guidelines, clinicians should negotiate with patients and their carers as early as possible on how information will be shared [﻿27﻿]. NICE guidelines emphasize the need to check how information is shared regularly, especially when communication difficulties are likely to occur. These aspects need to be fostered in order to improve adherence to both pharmacological and non-pharmacological treatments proposed. In particular, when considering pharmacological treatments, it is essential to evaluate the patient’s preferences, expectations and worries about the treatment in order to develop a personalized treatment strategy [57]. The adoption and the implementation of SDM for psychiatric medication management in the clinical routine care represents a big challenge for both mental health professionals as well as for users and carers.

## Suggestions for clinical practice

In recent years, considerable resources have been invested to make the SDM a routine way of working: computerized programs, role-play techniques, training groups for mental health staff [58]. The main target of these interventions are patients with schizophrenia and psychotic disorders.

SDM can be promoted in several ways: either by having the patient complete questionnaires during hospitalization to express their opinion regarding the satisfaction with the treatment received or through online platforms in which patients can express their treatment preferences, even before seeing their doctor—in a positive perspective of an active involvement of patients in the treatment of their mental illness.

The introduction of specific smartphone apps has led to the dissemination of a SDM protocol, but it must be considered that the use of apps requires specific skills. Therefore, it is likely that only patients with high level of personal functioning and less severe symptomatology can use these support tools, gaining a positive reinforcement [59, 60].

Based on the results of the eight studies included in this literature review, interventions focused on enhancement of the adoption in SDM in the clinical routine care seem promising, although these results are preliminary and only the short-term efficacy of these approaches has been confirmed. The interventions proposed appear feasible and well-accepted by both patients and clinicians, confirming the findings from the ROAMER study which showed that all stakeholders of mental health want to be actively involved in the planning and management of care [61, 62]. Before developing SDM interventions aiming to improve the acceptance rate of LAIs in patients with schizophrenia, further longitudinal methodologically rigorous studies are needed.

However, as pointed out by Das et al. [63﻿], patients’ and clinicians’ attitudes towards LAIs are a critical element in their underutilization in the clinical practice. Psychiatrists generally believe that patients are less willing to accept LAIs, than oral treatments and they avoid proposing it [64]. In fact, in the study by Kane et al. [﻿35﻿], when clinicians are adequately informed on how to appropriately manage it, the patients’ acceptance rate increases. In a qualitative study with young patients with psychosis, Das et al. [63﻿] found that patients prefer LAIs, since they do not have to remember to take pills every day.

Other factors hampering the underutilization of LAIs in clinical routine care include the overestimation of patient’s adherence; the time-consuming process of using SDM style for proposing the LAIs treatment; the heterogeneity of international guidelines and the prejudice that therapeutic relationship would be weakened by the adoption of LAIs, particularly in the early stages of the disease [65].

Therefore, international and national scientific associations should clearly state the potential beneficial role of using LAIs in the early stage of the disorder, supporting the use of LAIs and SDM style in proposing the switch from oral to LAI formulation to patients with schizophrenia.

It is essential that psychiatrists introduce the use of SDM into their clinical practice, not limiting themselves to accepting the patient’s preferences, but providing clear and comprehensive information [66]. The SDM seems to be particularly effective in the case of LAIs prescription, since patient have prejudices and fears related to the formulation, which the doctor must contrast [﻿35﻿]. At the same time, the active involvement of the patient in the discussion on the type of formulation to be adopted can be useful to reduce the perceived coercion reported by patients in receiving pharmacological treatments. The current and future increasing availability of LAIs will enrich the choice for the clinicians who intend to use a long-acting formulation [67]. Some effective strategies to actively involve patients in the discussion about the type of LAI include to discuss his/her fear about the injection procedure, discussing previous negative personal experiences with LAI medications, describe the positive effects of such formulation, evaluate the level of patient’s motivation towards the pharmacological treatment. Possible recommendations for best clinical practice and on how to propose to start a treatment with LAIs are summarized in Table 2.

**Table 2:** Recommendations and positional statements in proposing LAI antipsychotics in the management of patients with schizophrenia

Use a SDM-based approach, informing the patient in a clear and simple way and accepting his/her requests
Be welcoming towards patients who are afraid of needles, without minimizing their fear
If the patient has had previous negative experiences with another LAI antipsychotic, reassure him/her and explain clearly why it is desirable to start the new LAI antipsychotic
Emphasize to the patient that he/she will not have to take oral therapy, will no longer have the risk of forgetting it and the annoyance of carrying tablets along
Communicate that LAI antipsychotics are better than oral drugs in preventing relapses and re-hospitalizations
Propose LAI antipsychotics in the early stages of illness, explaining that a stable therapy is associated with a better outcome
Involve family members and caregivers in the decision process

*LAI* long acting injection, *SDM* shared decision making

Involving patients in the choice of therapy is not sufficient to increase pharmacological adherence if, at the same time, there is no constant work of comparison and communication with the reference psychiatric team. Therefore, in our opinion, resources should be allocated for health personnel dedicated training and to support patients, for example with ad hoc courses (e.g., TEMPO training). The results will then be monitored over time in order to evaluate the impact of these interventions on the recovery rates in patients with schizophrenia.
